# Supplementary material for: Early alveolar macrophage response and IL-1R-dependent T cell priming determine transmissibility of Mycobacterium tuberculosis strains
Source: Nat Commun. 2022 Feb 16;13:884. doi: 10.1038/s41467-022-28506-2 (PMC8850437; doi:10.1038/s41467-022-28506-2)
Supplement: Supplementary file 1 — Supplementary Information [file 41467_2022_28506_MOESM1_ESM.pdf]

Supplementary information for:

Early alveolar macrophage response and IL-1R-dependent T cell Priming determine transmissibility of *Mycobacterium tuberculosis* Strains

Arianne Lovey[1], Sheetal Verma[1], Vaishnavi Kaipilyawar[1], Rodrigo Ribeiro-Rodrigues[2], Seema Husain[3], Moises Palaci[2], Reynaldo Dietze[4], Shuyi Ma[4,5,6,7], Robert D Morrison[8], David. R. Sherman[9], Jerrold J. Ellner[1] and Padmini Salgame[1]*

[1]Center for Emerging Pathogens, Department of Medicine, Rutgers-New Jersey Medical School, Newark, NJ, USA.

[2]Núcleo de Doenças Infecciosas – UEFS, Vitoria, Brazil.

[3]The Genomics Center, Rutgers- New Jersey Medical School, Newark, NJ, USA,

[4]Global Health & Tropical Medicine - Instituto de Higiene e Medicina Tropical - Universidade Nova de Lisboa, Lisbon, Portugal.

[4]Center for Global Infectious Disease Research, Seattle Children's Research Institute, Seattle WA.

[5]Division of Infectious Diseases, Department of Pediatrics, University of Washington, Seattle WA.

[6]Pathobiology Program, Department of Global Health, University of Washington, Seattle WA.
[7]Department of Chemical Engineering, University of Washington, Seattle WA.

[8]Laboratory of Malaria Immunology and Vaccinology, National Institute of Allergy and Infectious Diseases, NIH, Bethesda, Maryland, USA.

[9]Department of Microbiology, University of Washington, Seattle, WA 98109-8070

*Corresponding Author Padmini Salgame, Rutgers - New Jersey Medical School, Email: padmini.salgame@rutgers.edu

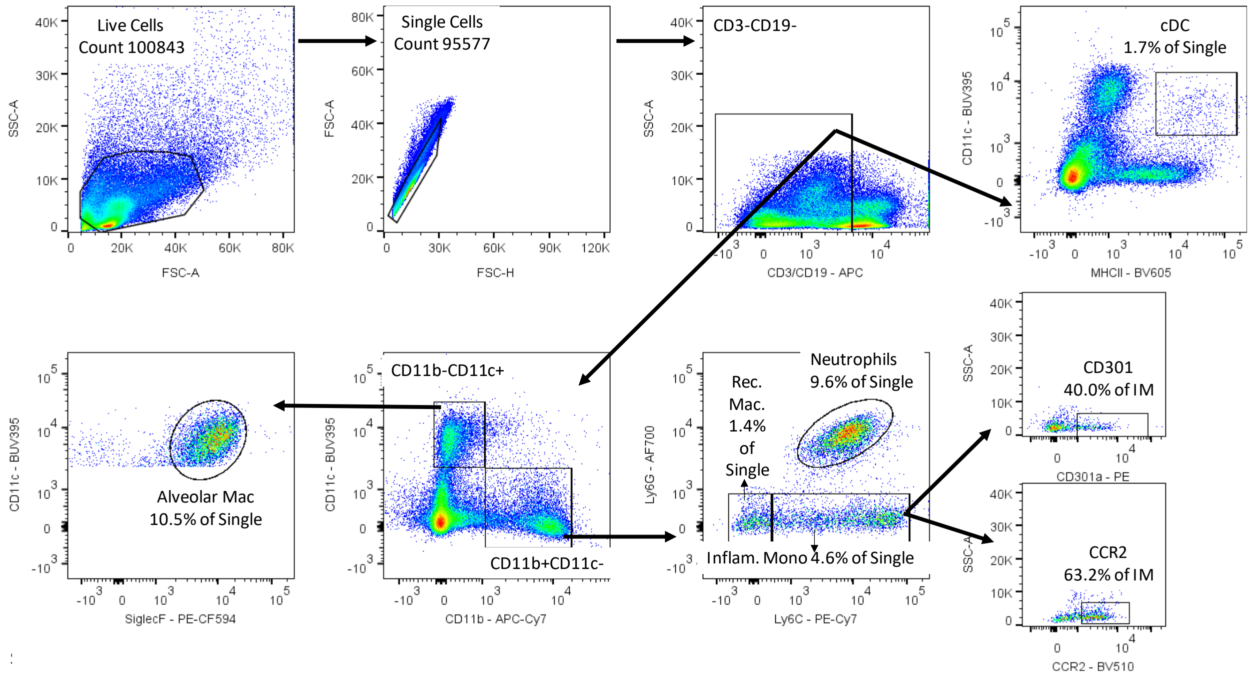

Supplementary Figure 1. Gating strategy for flow cytometric analysis of lung cellular infiltrates. Live, single cells were identified using SSC-A, FSC-A and FSC-A. Myeloid cells were identified as the CD3$^-$ and CD19- population. From the CD3$^-$CD19$^-$ population, CD11c and MHCII expressing cells were gated out as cDCs and CD11c+, CD11b- were gated out and marked as AM if positive for SiglecF expression. The CD11b+c- cells were segregated as recruited macrophages if Ly6G- and Ly6C-, neutrophils if Ly6G+ and as inflammatory monocytes if Ly6C+. The CD11b+c-Ly6+ cells were further separated as CD301+ and CCR2+ interstitial macrophages. Dendritic cells CD3-CD19-CD11b+CD11c+; Alveolar macrophages CD3-CD19-CD11b-CD11c+SiglecF+; Recruited macrophages CD3-CD19-CD11b+CD11c-Ly6c-Ly6g-; Neutrophils CD3-CD19-CD11b+CD11c-Ly6c+Ly6g+; Inflammatory monocytes CD3-CD19-CD11b+CD11c-Ly6c+Ly6g-. Inflammatory monocyte subsets were either CCR2+ or CD301a+.

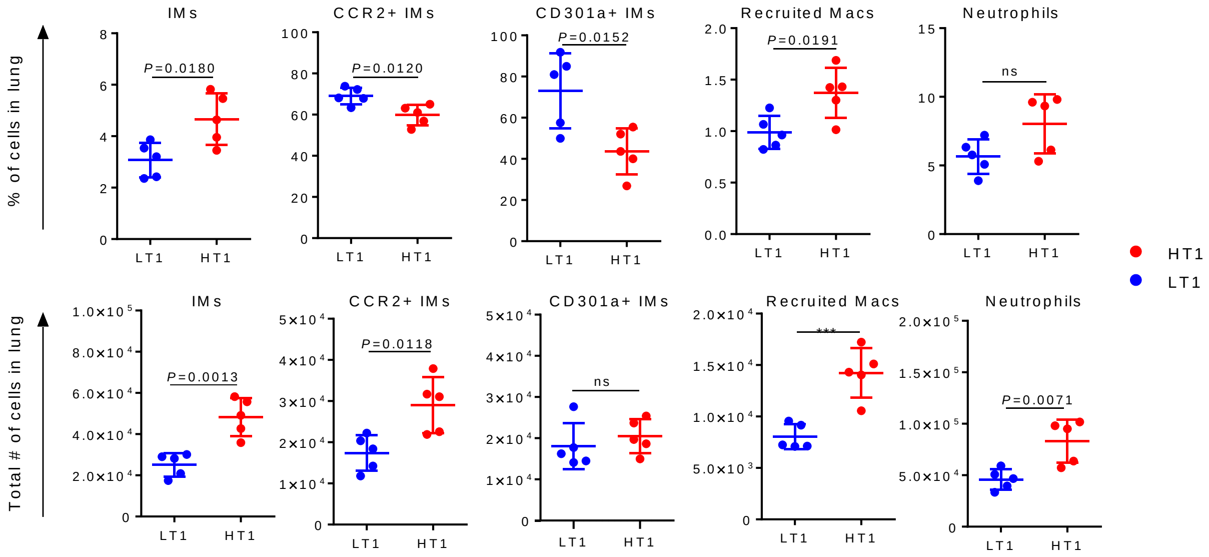

Supplementary Figure 2. C3HeB/FeJ mice were infected with ~100 CFU of the indicated strain via Glas-Col aerosol exposure. At the indicated time post infection mice were sacrificed the right middle and inferior lung lobes were isolated to obtain single cells. Percent and total number of neutrophils (CD3-CD19-CD11b+CD11c-Ly6C+Ly6G+), recruited macrophages (CD3-CD19-CD11b+CD11c-Ly6C-Ly6G- cells), and inflammatory monocytes (CD3-CD19-CD11b+CD11c-Ly6C+Ly6G-) including CCR2+ and CD301a+ subsets were calculated following acquisition on LSRFortessa X-20 and FlowJo analysis. Sample size of n=5 mice was examined in each group. Data are presented as mean values +/- SD. Source data are provided as a Source Data file. Significance was determined using unpaired t test.

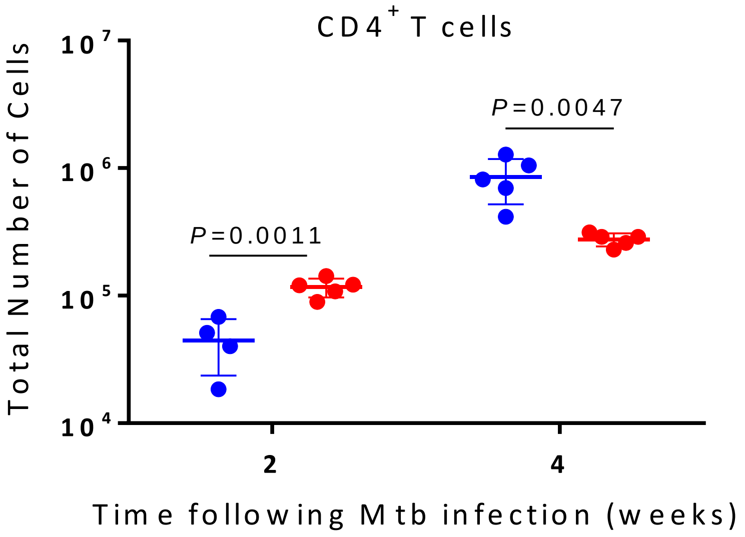

Supplementary Figure 3. C3HeB/FeJ mice were infected with ~100 CFU and at the indicated time post infection mice were sacrificed and lung lobes harvested, following which single cell suspensions were prepared. Total $CD4^+$ T cells was determined by acquisition on LSRFortessa X-20. Sample size of n=5 mice was included in each group. Significance was determined using using unpaired t test one-way ANOVA with Tukey's correction. $**p<0.01$.

Supplementary Table 1

Mtb Strain	NC_000962 (Gene)	locus_tag (NC_000962 (Gene))	Region	Type	Reference	Allele	Length	Repeat region	Product	Note	GeneID (NC_000962)	Protein id
Mtb-LT1	gyrB	Rv0005	6140	SNV	G	T	1		DNA gyrase subu	Belongs to the ty	GeneID:887081	NP_214519.2
Mtb-HT1			13636	SNV	T	C	1					
Mtb-LT1			34149	SNV	G	C	1					
Mtb-LT1	Rv0039c	Rv0039c	42014	SNV	G	C	1		transmembrane p	A core mycobacte	GeneID:887038	NP_214553.1
Mtb-LT1	Rv0039c	Rv0039c	42350	SNV	A	G	1		transmembrane p	A core mycobacte	GeneID:887038	NP_214553.1
Mtb-LT1	Rv0045c	Rv0045c	49439	SNV	C	T	1		hydrolase		GeneID:887029	NP_214559.1
Mtb-LT1	Rv0045c	Rv0045c	49764	SNV	C	A	1		hydrolase		GeneID:887029	NP_214559.1
Mtb-HT1	ponA1	Rv0050	54786	SNV	G	A	1		bifunctional peni	penicillin-binding	GeneID:887065	
Mtb-LT1	ssb	Rv0054	58808	SNV	G	A	1		single-strand DNA-binding protein		GeneID:887013	NP_214568.1
Mtb-LT1	Rv0079	Rv0079	88333	SNV	G	A	1		hypothetical prot	possible vaccine	GeneID:886995	NP_214593.1
Mtb-LT1	ctpA	Rv0092	100767	SNV	A	G	1		cation transporte	Belongs to the ca	GeneID:886946	NP_214606.1
Mtb-LT1	Rv0094c	Rv0094c	103756	SNV	G	T	1	REP-2, len: 1503 nt. REP251, member of REP13E12family.	hypothetical protein		GeneID:886943	NP_214608.1
Mtb-HT1	Rv0095c	Rv0095c	104962	SNV	G	A	1	REP-2, len: 1503 nt. REP251, member of REP13E12family.	hypothetical protein		GeneID:886940	NP_214609.1
Mtb-LT1	Rv0095c	Rv0095c	105060	SNV	G	A	1	REP-2, len: 1503 nt. REP251, member of REP13E12family.	hypothetical protein		GeneID:886940	NP_214609.1
Mtb-LT1	Rv0095c	Rv0095c	105063	SNV	G	A	1	REP-2, len: 1503 nt. REP251, member of REP13E12family.	hypothetical protein		GeneID:886940	NP_214609.1
Mtb-LT1	fadD10	Rv0099	108572	SNV	G	A	1		fatty-acid--CoA ligase FadD10		GeneID:886933	NP_214613.1
Mtb-HT1	PE_PGRS1	Rv0109	132417	SNV	C	G	1		PE-PGRS family p	Member of the M	GeneID:886912	
Mtb-LT1	Rv0110	Rv0110	133445	SNV	G	A	1		integral membrane protein		GeneID:886917	NP_214624.1
Mtb-LT1	Rv0122	Rv0122	148652	SNV	C	T	1		hypothetical protein		GeneID:886888	NP_214636.1
Mtb-LT1	PE_PGRS2	Rv0124	150890	SNV	A	G	1		PE-PGRS family p	Member of the M	GeneID:886883	
Mtb-HT1	Rv0149	Rv0149	175905	SNV	T	C	1		quinone oxidored	belongs to the zir	GeneID:886843	NP_214663.1
Mtb-HT1	mce1D	Rv0172	202961	SNV	A	G	1		Mce family protein Mce1D		GeneID:886807	NP_214686.1
Mtb-LT1	mce1F	Rv0174	205767	SNV	C	T	1		Mce family protein Mce1F		GeneID:886820	NP_214688.1
Mtb-HT1	Rv0181c	Rv0181c	212813	SNV	T	C	1		hypothetical protein		GeneID:886788	NP_214695.1
Mtb-LT1			218637	SNV	T	C	1					
Mtb-LT1	Rv0194	Rv0194	228168	SNV	G	C	1		multidrug ABC tra	Belongs to the AT	GeneID:886790	NP_214708.1
Mtb-HT1	mmpL3	Rv0206c	244565	SNV	C	T	1		transmembrane transport protein M		GeneID:886752	NP_214720.1
Mtb-HT1	lipW	Rv0217c	259997	SNV	C	T	1		esterase LipW		GeneID:886726	NP_214731.1
Mtb-LT1	Rv0218	Rv0218	261562	SNV	G	A	1		transmembrane protein		GeneID:886727	NP_214732.1
Mtb-HT1	Rv0221	Rv0221	265219	SNV	C	T	1		diacyglycerol O-a	triacylglycerol syr	GeneID:886719	NP_214735.1
Mtb-HT1	Rv0226c	Rv0226c	269914	SNV	C	A	1		transmembrane p	A core mycobacte	GeneID:886711	NP_214740.1
Mtb-HT1	fadE4	Rv0231	277201	SNV	C	T	1		acyl-CoA dehydrogenase FadE4		GeneID:886703	NP_214745.1
Mtb-LT1	nrdB	Rv0233	279006	SNV	A	T	1		ribonucleoside-d	Belongs to the rib	GeneID:886699	NP_214747.1
Mtb-HT1	fadE5	Rv0244c	295009	SNV	T	C	1		acyl-CoA dehydrogenase FadE5		GeneID:886698	NP_214758.1
