# Supplementary material for: Early alveolar macrophage response and IL-1R-dependent T cell priming determine transmissibility of Mycobacterium tuberculosis strains
Source: Nat Commun. 2022 Feb 16;13:884. doi: 10.1038/s41467-022-28506-2 (PMC8850437; doi:10.1038/s41467-022-28506-2)
Supplement: Supplementary file 3 — Reporting Summary [file 41467_2022_28506_MOESM3_ESM.pdf]

nature portfolio

Corresponding author(s): Padmini Salgame

Last updated by author(s): Jan 26, 2022

# Reporting Summary

Nature Portfolio wishes to improve the reproducibility of the work that we publish. This form provides structure for consistency and transparency in reporting. For further information on Nature Portfolio policies, see our Editorial Policies and the Editorial Policy Checklist.

## Statistics

For all statistical analyses, confirm that the following items are present in the figure legend, table legend, main text, or Methods section.

| n/a | Confirmed | |
|---|---|---|
| ☐ | ☒ | The exact sample size (*n*) for each experimental group/condition, given as a discrete number and unit of measurement |
| ☐ | ☒ | A statement on whether measurements were taken from distinct samples or whether the same sample was measured repeatedly |
| ☐ | ☒ | The statistical test(s) used AND whether they are one- or two-sided<br>*Only common tests should be described solely by name; describe more complex techniques in the Methods section.* |
| ☒ | ☐ | A description of all covariates tested |
| ☐ | ☒ | A description of any assumptions or corrections, such as tests of normality and adjustment for multiple comparisons |
| ☐ | ☒ | A full description of the statistical parameters including central tendency (e.g. means) or other basic estimates (e.g. regression coefficient) AND variation (e.g. standard deviation) or associated estimates of uncertainty (e.g. confidence intervals) |
| ☐ | ☒ | For null hypothesis testing, the test statistic (e.g. *F*, *t*, *r*) with confidence intervals, effect sizes, degrees of freedom and *P* value noted<br>*Give P values as exact values whenever suitable.* |
| ☒ | ☐ | For Bayesian analysis, information on the choice of priors and Markov chain Monte Carlo settings |
| ☒ | ☐ | For hierarchical and complex designs, identification of the appropriate level for tests and full reporting of outcomes |
| ☒ | ☐ | Estimates of effect sizes (e.g. Cohen's *d*, Pearson's *r*), indicating how they were calculated |

*Our web collection on statistics for biologists contains articles on many of the points above.*

## Software and code

Policy information about availability of computer code

| | |
|---|---|
| Data collection | Statistical Graphing Software GraphPad Prism 9 was used. |
| Data analysis | 1. MesoScale Discovery WORKBENCH 4.0.12 (LSR 4-0-12) analysis software; 2. Flowjo Software (Tree Star, Inc.) v10.6.1; 3. NanoString nSolver 4 software, DEG analysis using NanoStringDiff (R package version 1.22.0); 4. Bioconductor version release 3.13; 5. Brightfield 40x histology slide scans analysis on Leica SCN 400F whole slide scanner. |

For manuscripts utilizing custom algorithms or software that are central to the research but not yet described in published literature, software must be made available to editors and reviewers. We strongly encourage code deposition in a community repository (e.g. GitHub). See the Nature Portfolio guidelines for submitting code & software for further information.

## Data

Policy information about availability of data

All manuscripts must include a data availability statement. This statement should provide the following information, where applicable:

- Accession codes, unique identifiers, or web links for publicly available datasets
- A description of any restrictions on data availability
- For clinical datasets or third party data, please ensure that the statement adheres to our policy

- Data is available through this link. https://dataview.ncbi.nlm.nih.gov/object/PRJNA759569?reviewer=1cjilf6grq8huvp1g95p7dguv6.
- Additional data supporting the findings of this study are available in Supplementary Information. Source Data file is included in Supplementary Information.

# Field-specific reporting

Please select the one below that is the best fit for your research. If you are not sure, read the appropriate sections before making your selection.

☒ Life sciences ☐ Behavioural & social sciences ☐ Ecological, evolutionary & environmental sciences

For a reference copy of the document with all sections, see nature.com/documents/nr-reporting-summary-flat.pdf

# Life sciences study design

All studies must disclose on these points even when the disclosure is negative.

| | |
|---|---|
| Sample size | Sample sizes are indicated in the manuscript. No sample size calculation was performed. Sample sizes were based on accepted standards in the field and based on our prior study (PMID: 30840702). |
| Data exclusions | Outliers were identified using the Grubb's test in GraphPad Prism software. Data points were excluded in Figures 4c, 5b, 5c, 6b, 6c since they were identified as outliers. Exclusion criteria were pre-established. |
| Replication | Experimental findings were replicated in independent experiments and the exact number is provided in the figure legends. All attempts at replication were successful. |
| Randomization | C3HeB/FeJ mice were purchased from The Jackson Laboratory and divided in groups of same age and sex. Randomization was not necessary since assays had a quantitative readout eliminating user bias. |
| Blinding | Largely blinding was not necessary because data are reported as quantitative measurements. Histopathological evaluation of lung sections was performed by an investigator blinded to the experimental groups. |

# Reporting for specific materials, systems and methods

We require information from authors about some types of materials, experimental systems and methods used in many studies. Here, indicate whether each material, system or method listed is relevant to your study. If you are not sure if a list item applies to your research, read the appropriate section before selecting a response.

## Materials & experimental systems

| n/a | Involved in the study | |
|---|---|---|
| ☐ | ☒ | Antibodies |
| ☒ | ☐ | Eukaryotic cell lines |
| ☒ | ☐ | Palaeontology and archaeology |
| ☐ | ☒ | Animals and other organisms |
| ☒ | ☐ | Human research participants |
| ☒ | ☐ | Clinical data |
| ☒ | ☐ | Dual use research of concern |

## Methods

| n/a | Involved in the study | |
|---|---|---|
| ☒ | ☐ | ChIP-seq |
| ☐ | ☒ | Flow cytometry |
| ☒ | ☐ | MRI-based neuroimaging |

## Antibodies

| | |
|---|---|
| Antibodies used | The following antibodies were used (in all cases lot numbers were not recorded)- anti-mouse CD3-APC (clone 17A2; BD Pharmingen 565643), anti-mouse CD19-APC (clone 1D3, BD Pharmingen 561738), anti-mouse CD11b-APC-Cy7 (clone M1/70; BD Pharmingen 557657), anti-mouse CD11c-BUV395 (clone HL3; BD Horizon 564080), anti-mouse Ly6G-AF700 (clone 1A8; BD Pharmingen 561236), anti-mouse Ly6C-PECy7 (clone AL-21; BD Pharmingen 560593), anti-mouse SiglecF-PE-CF594 (clone E50-2550; BD Horizon 562757), anti-mouse MHC-II (clone M5/114.15.2; BD Horizon 563413), anti-mouse CCR2 (clone SA203G11; Biolegend 150617) and anti-mouse CD301a (clone LOM-8.7; Biolegend 145606), anti-mouse CD3-FITC (clone 17A2, BD Pharmingen 555274), anti-mouse CD4-V450 (clone RM4-5, BD Horizon 560468), and anti-mouse CD8-PE (clone RM4-5, BD Pharmingen 553033), anti-mouse IL-17A-AF647 (clone TC11-18H10, BD Pharmingen 560184) and IFNγ-PECy7 (clone XMG1.2, BD Pharmingen 557649). Antibodies to IL-1R (Bio X Cell BE0256) and Armenian hamster isotype control IgG (Bio X Cell BE0091) were used for IL-1 blocking experiments. |
| Validation | All antibodies are routinely tested by the manufacturers with positive and negative controls as shown. Compensation beads (BDB552845) were used with each antibody at the time of experiment as single color controls. |

## Animals and other organisms

Policy information about studies involving animals; ARRIVE guidelines recommended for reporting animal research

| | |
|---|---|
| Laboratory animals | 5–7-week-old female C3HeB/FeJ mice (Jackson #000658) were used in this study. Mice are housed at ambient temperature set at 72 degrees +/- 4 degrees, humidity range 35-70 and 12-hour light dark cycle (7 am on 7 pm off). |

| | |
|---|---|
| Wild animals | Were not used in this study. |
| Field-collected samples | Were not used in this study. |
| Ethics oversight | The studies with mice described here conform to the Rutgers-NJMS Institutional Animal Care and Use Committee (IACUC) Guidelines, NIH and USDA policies on the care and use of animals in research. Animal protocols pertaining to the experiments in the study were approved by Rutgers IACUC. |

Note that full information on the approval of the study protocol must also be provided in the manuscript.

# Flow Cytometry

## Plots

Confirm that:

☒ The axis labels state the marker and fluorochrome used (e.g. CD4-FITC).

☒ The axis scales are clearly visible. Include numbers along axes only for bottom left plot of group (a 'group' is an analysis of identical markers).

☒ All plots are contour plots with outliers or pseudocolor plots.

☒ A numerical value for number of cells or percentage (with statistics) is provided.

## Methodology

| | |
|---|---|
| Sample preparation | All cells were obtained from female C3HeB/FeJ mice. Lung lobes were digested with 20µg/ml of collagenase D at 37o C for 30 min, and reaction was stopped by the addition of 5mM of EDTA. Digested lung tissue was passed through 40µm nylon membrane filter. Spleen and lymph nodes were passed through a 40µm nylon membrane filter directly. Red blood cells were lysed by treating with ACK (Ammonium-chloride-Potassium) lysing buffer (Quality Biological 118-156-101) and washed with PBS to obtain single cells. |
| Instrument | Samples were acquired using the BD Biosciences LSRFortesso X-20. |
| Software | Samples were analyzed using Flow Jo, v.10. Raw numbers for each group were exported to excel. Following additional calculations, GraphPad Prism was used to plot and statistically analyze the percent and total number of the selected populations. |
| Cell population abundance | The number of single cells (identified as those cells with appropriate SSC-A, FSC-A and FSC-H parameters in Flow Jo, (as shown) was used to calculate the percent of population abundance for each cell population. Total number of cells for each subpopulation was then calculated using the percentage obtained as described and then multiplied by the calculated number of cells for each sample as counted under the microscope at a known dilution. |
| Gating strategy | SSC-A and FSC-A parameters were used to identify live cells with cellular debris (bottom right corner and top left arc) removed. Following the identification of live cells, doublets were removed, and single cells were selected based on FSC-A and FSC-H as indicated. The single cells as selected using FSC-A and FSC-H accounted for 97-99% of live cells as selected in the previous gate. From here, either CD3 and CD19 or CD4, CD8 and B220 were used as negative selection markers. Double (or triple) negative cells were then gated based on either CD11c and MHCII or CD11b and CD11c expression. CD3-CD19-CD11b +CD11c+MHCII+ cells were identified to be cDCs with CD11b+ and CD103+ subsets. Then, CD3-CD19-CD11b-CD11c+ population was evaluated for SiglecF expression and used to identify alveolar macrophages. The CD11bCd11c double positive population was selected and further analyzed based on Ly6C and Ly6G expression. From here, neutrophils were defined as CD3-CD19-CD11b+CD11c-Ly6C+Ly6G+ cells, Inflammatory monocytes were defined as CD3-CD19-CD11b+CD11c-Ly6C+Ly6G- cells with CCR2+ and CD301a+ subsets, and Recruited macrophages were defined as CD3-CD19-CD11b+CD11c-Ly6C-Ly6G- cells. Single color controls for each marker and FMOs for CD11c, CD11b, Ly6C and Ly6G were used to identify the boundaries between positive and negative staining cell populations in each experiment. |

☒ Tick this box to confirm that a figure exemplifying the gating strategy is provided in the Supplementary Information.
